# The Structure of Character Strengths: Variable- and Person-Centered Approaches

**DOI:** 10.3389/fpsyg.2018.00153

**Published:** 2018-02-20

**Authors:** Małgorzata Najderska, Jan Cieciuch

**Affiliations:** ^1^Institute of Psychology, Faculty of Christian Philosophy, Cardinal Stefan Wyszynski University in Warsaw, Warsaw, Poland; ^2^University Research Priority Program Social Networks, University of Zurich, Zurich, Switzerland

**Keywords:** character strengths, variable-centered approach, person-centered approach, personality traits, metatraits

## Abstract

This article examines the structure of character strengths (Peterson and Seligman, 2004) following both variable-centered and person-centered approaches. We used the International Personality Item Pool-Values in Action (IPIP-VIA) questionnaire. The IPIP-VIA measures 24 character strengths and consists of 213 direct and reversed items. The present study was conducted in a heterogeneous group of *N* = 908 Poles (aged 18–78, *M* = 28.58). It was part of a validation project of a Polish version of the IPIP-VIA questionnaire. The variable-centered approach was used to examine the structure of character strengths on both the scale and item levels. The scale-level results indicated a four-factor structure that can be interpreted based on four of the five personality traits from the Big Five theory (excluding neuroticism). The item-level analysis suggested a slightly different and limited set of character strengths (17 not 24). After conducting a second-order analysis, a four-factor structure emerged, and three of the factors could be interpreted as being consistent with the scale-level factors. Three character strength profiles were found using the person-centered approach. Two of them were consistent with alpha and beta personality metatraits. The structure of character strengths can be described by using categories from the Five Factor Model of personality and metatraits. They form factors similar to some personality traits and occur in similar constellations as metatraits. The main contributions of this paper are: (1) the validation of IPIP-VIA conducted in variable-centered approach in a new research group (Poles) using a different measurement instrument; (2) introducing the person-centered approach to the study of the structure of character strengths.

## Introduction

Peterson and Seligman (2004) have proposed the extension of personality traits research by introducing the concepts of character strengths and virtues. The handbook for the theory, *Character Strengths and Virtues: A Handbook and Classification* (also called by the authors *Manual of the Sanities*) catalogs these concepts by analogy to the disorders that are classified in the *Diagnostic and Statistical Manual of Mental Disorders* (Peterson and Seligman, 2004). After analyzing the world's most dominant intellectual and spiritual traditions (e.g., Judeo-Christianity, Athenian Greece, Islam, Confucianism, Taoism, Buddhism, Hinduism), Peterson and Seligman (2004) distinguished six common virtues which are defined as, “the core human characteristics valued by moral philosophers and religious thinkers” (p. 13). Character strengths are specific, measurable aspects of virtues, and are defined as “psychological ingredients–processes or mechanisms–that define virtues” (Peterson and Seligman, 2004, p. 13). Operationalizations of character strengths usually consider them as interpersonal differences–dimensions that describe the intensity of these processes, as defined by Peterson and Seligman (2004).

There is one catalog of character strengths and virtues but two similar, alternative lists that designate character strengths: the first is by Peterson and Seligman (2004) and the second is offered by Goldberg in the framework of the International Personality Item Pool (IPIP) project (Goldberg, 1999; Goldberg et al., 2006), which was formulated to better include the content of the items selected to measure character strength within this project. Table 1 presents the two lists of character strengths with short descriptions of each one. Because the use of two catalogs can be confusing, in this article, we use the character strength names that are common to both lists (in italics).

**Table 1:** Lists of virtues, their corresponding character strengths (Peterson and Seligman, 2004; Goldberg et al., 2006, IPIP project), and short descriptions of the character strengths (Park and Peterson, 2010, pp. 540–541).

**Virtues and character strengths (Peterson and Seligman, 2004)**	**Virtues and character strengths in the IPIP project (Goldberg et al., 2006)**	**Descriptions of character strengths**
**WISDOM/KNOWLEDGE**	**WISDOM**	
*Creativity* [originality, ingenuity]	Originality/*creativity*	Thinking of novel and productive ways to conceptualize and do things; includes artistic achievement but is not limited to it
*Curiosity* [interest, novelty-seeking, openness to experience]	*Curiosity*	Taking an interest in ongoing experience for its own sake; finding subjects and topics fascinating; exploring and discovering
Open-mindedness [*judgment*, critical thinking]	*Judgment*/open-mindedness	Thinking things through and examining them from all sides; not jumping to conclusions; being able to change one's mind in light of evidence; weighing all evidence fairly
*Love of learning*	*Love of learning*	Mastering new skills, topics, and bodies of knowledge, whether on one's own or formally; related to the strength of “curiosity” but goes beyond it to describe the tendency to add systematically to what one knows
*Perspective* [wisdom]	*Perspective*/wisdom	Being able to provide wise counsel to others; having ways of looking at the world that make sense to oneself and to other people
**COURAGE**	**COURAGE**	
*Bravery* [valor]	Valor/*bravery*	Not shrinking from threat, challenge, difficulty, or pain; speaking up for what is right even if there is opposition; acting on convictions even if unpopular; includes physical bravery but is not limited to it
*Persistence* [perseverance, industriousness]	Industry/perseverance/*persistence*	Finishing what one starts; persisting in a course of action in spite of obstacles; “getting it out the door”; taking pleasure in completing tasks
Integrity [authenticity, *honesty*]	Integrity/*honesty*/authenticity	Speaking the truth and more broadly presenting oneself in a genuine way and acting in a sincere way; being without pretense; taking responsibility for one's feelings and actions
Vitality [*zest*, enthusiasm, vigor, energy]	*Zest*/enthusiasm/vitality	Approaching life with excitement and energy; not doing things halfway or halfheartedly; living life as an adventure; feeling alive and activated
**HUMANITY**	**HUMANITARY**	
*Love*	Capacity for *love*	Valuing close relations with others, in particular those in which sharing and caring are reciprocated; being close to people
*Kindness* [generosity, nurturance, care, compassion, altruistic love, “niceness”]	*Kindness*/generosity	Doing favors and good deeds for others; helping them; taking care of them
*Social intelligence* [emotional intelligence, personal intelligence]	*Social*/personal/emotional *intelligence*	Being aware of the motives and feelings of other people and oneself; knowing what to do to fit into different social situations; knowing what makes other people tick
**JUSTICE**	**JUSTICE**	
Citizenship [social responsibility, loyalty, *teamwork*]	Citizenship/*teamwork*	Working well as a member of a group or team; being loyal to the group; doing one's share
*Fairness*	Equity/*fairness*	Treating all people the same according to notions of fairness and justice; not letting personal feelings bias decisions about others; giving everyone a fair chance
*Leadership*	*Leadership*	Encouraging a group of which one is a member to get things done and at the same time maintain good relations within the group; organizing group activities and seeing that they happen
**TEMPERANCE**	**TEMPERANCE**	
*Forgiveness* and mercy	*Forgiveness*/mercy	Forgiving those who have done wrong; accepting the shortcomings of others; giving people a second chance; not being vengeful
Humility and *modesty*	*Modesty*/humility	Letting one's accomplishments speak for themselves; not seeking the spotlight; not regarding oneself as more special than one is
*Prudence*	*Prudence*	Being careful about one's choices; not taking undue risks; not saying or doing things that might later be regretted
*Self-regulation* [self-control]	*Self-regulation*/self-control	Regulating what one feels and does; being disciplined; controlling one's appetites and emotions
**TRANSCENDENCE**	**TRANSCENDENCE**	
Appreciation of *beauty* and excellence [awe, wonder, elevation]	Appreciation for *beauty*	Noticing and appreciating beauty, excellence, and/or skilled performance in various domains of life, from nature to art to mathematics to science to everyday experience
*Gratitude*	*Gratitude*	Being aware of and thankful for the good things that happen; taking time to express thanks
*Hope* [optimism, future-mindedness, future-orientation]	*Hope*/optimism	Expecting the best in the future and working to achieve it; believing that a good future is something that can be brought about
*Humor* [playfulness]	*Humor*/playfulness	Liking to laugh and joke; bringing smiles to other people; seeing the light side; making (not necessarily telling) jokes
*Spirituality* [religiousness, faith, purpose]	*Spirituality*/religiousness	Having coherent beliefs about the higher purpose and meaning of the universe; knowing where one fits within the larger scheme; having beliefs about the meaning of life that shape conduct and provide comfort

*Virtues are written in capital letters. The common elements of the character strengths from both catalogs are italicized. These names will be used in this article*.

Researchers across the globe have demonstrated the usefulness of the character strengths construct for explaining several aspects of human functioning such as well-being (Wood et al., 2011), school/academic achievement (Shoshani and Slone, 2013), positive work experience (Harzer and Ruch, 2012), or life satisfaction (Park et al., 2004). However, the large catalog of 24 character strengths concurrently raises questions about relations between them and about their structure. We consider the structure of character strengths as a valid issue for three reasons. First, 24 variables are a lot, and they most likely are not orthogonal. Therefore, we should examine the possible (and probable) overlap between them. Second, we consider parsimony to be an important feature of every psychological theory so it is always worthwhile to look for a simplified structure. Finally, by analogy to the research in the lexical domain (where many smaller traits are gathered under the “umbrella” of the Big Five traits), we wanted to specifically investigate what factors are formed by which character strengths.

To answer the questions about the structure, researchers often use exploratory factor analysis both on scales (Macdonald et al., 2008; Peterson et al., 2008; Brdar and Kashdan, 2010; Ruch et al., 2010; Shryack et al., 2010; Singh and Choubisa, 2010; Littman-Ovadia and Lavy, 2012; McGrath, 2014) or scales and items (McGrath, 2014; Ng et al., 2017). Latent structure was investigated by using the “bass-ackwards” procedure (McGrath, 2015).

A factor analysis of character strengths was outlined by Peterson and Seligman (2004). Despite the fact that they did not present a full description of the results, they identified five factors: *interpersonal strengths, emotional strengths, intellectual strengths, strengths of restraint*, and *theological strengths*. This solution did not replicate the division of character strengths into six virtues; however, Peterson and Seligman (2004) argued that the existence of six virtues is purely theoretical. Virtues demonstrate which character strengths should be cultivated to achieve the virtue cited in philosophical and religious literature. In statistical terms, virtues cannot be conceptualized as latent variables that are indicated by all character strengths related to this virtue, because the same virtue (e.g., courage) can be achieved by the exercise of different strengths (e.g., bravery or vitality). That is why a six-virtue catalog is not replicated in the empirical studies on character strengths structure. Instead, studies (e.g., Peterson and Seligman, 2004; Macdonald et al., 2008; McGrath, 2014) show that there are four or five factors that correspond to the Big Five personality traits rather than six virtues that are differentiated in spiritual and philosophical traditions. Despite several inter-study differences in the factor solution, there are also similarities. Table 2 presents a summary of the research on the structure of character strengths and describes them in terms of Peterson and Seligman's (2004) original taxonomy derived from the factor analysis. Several factors in Table 2 are described by combining two or more factor names from Peterson and Seligman's (2004) research. There was only one factor that we could not properly assign—*civic strengths—*from studies by Singh and Choubisa (2010) conducted in India. The characteristics that comprise the civic strengths factors are connected to interpersonal relationships, but there is already another factor that is named as such in their classification.

**Table 2:** Results from studies of the structure of character strengths interpreted using Peterson and Seligman's (2004) classification obtained from an exploratory factor analysis.

**Study**	**Macdonald et al., 2008**	**Peterson et al., 2008**	**Brdar and Kashdan, 2010**	**Ruch et al., 2010**	**Singh and Choubisa, 2010**	**Shryack et al., 2010**	**Littman-Ovadia and Lavy, 2012**	**McGrath, 2014**
Country	Australia	Online study	Croatia	Germany	India	USA	Israel	Online study
*Factor*	*Interpersonal strengths* (Positivity)	*Interpersonal*	*Interpersonal*	*Interpersonal strengths*	*Interpersonal strengths*	*Interpersonal strengths* (Sociability)	*Interpersonal strengths*	*Interpersonal*
Content	Teamwork	Humor	Fairness	Fairness	Humor	Kindness	Modesty	Fairness
	Love	Kindness	Teamwork	Teamwork	Social intelligence	Teamwork	Fairness	Kindness
	Hope	Leadership	Kindness	Forgiveness	Bravery	Love	Forgiveness	Teamwork
	Humor	Love	Forgiveness	Modesty	Kindness	Gratitude		Modesty
	Zest	Social intelligence	Love	Kindness		Fairness		Leadership
	Leadership	Teamwork	Modesty	Leadership		Leadership		Forgiveness
			Leadership			Forgiveness		
			Gratitude			Humor		
			Beauty					
*Factor*	*Emotional/Theological strengths* (Niceness)	*Emotional strengths/Strengths of restraint* (Fortitude)	*Emotional strengths* (Vitality)	*Emotional strengths*	*?* (Civic strengths)	*Intellectual/Interpersonal/ Emotional strengths* (Agency/Self-assuredness)	*Emotional strengths*	*Emotional*
Content	Modesty	Bravery	Zest	Humor	Fairness	Creativity	Love	Social intelligence
	Fairness	Honesty	Hope	Zest	Teamwork	Perspective	Kindness	Humor
	Kindness	Judgment	Curiosity	Love	Modesty	Social intelligence	Social intelligence	Bravery
	Forgiveness	Persistence	Humor	Hope	Leadership	Bravery	Humor	Creativity
	Spirituality	Perspective		Bravery	Honesty	Curiosity	Teamwork	Perspective
	Gratitude	Self-regulation		Social intelligence	Prudence	Hope	Leadership	
						Love of learning		
						Zest		
						Judgment		
						Beauty		
Factor	*Intellectual strengths* (Intellect)	*Intellectual strengths* (Cognitive)	*Intellectual strengths* (Fortitude)	*Intellectual strengths*	*Intellectual strengths*	*Intellectual strengths* (Conscientiousness)	*Intellectual strengths*	*Intellectual*
Content	Creativity	Beauty	Perspective	Love of learning	Creativity	Prudence	Love of learning	Love of learning
	Beauty	Creativity	Judgment	Creativity	Judgment	Self-regulation	Curiosity	Beauty
	Curiosity	Curiosity	Creativity	Curiosity	Love of learning	Persistence	Creativity	Curiosity
	Love of learning	Love of learning	Social intelligence	Judgment	Curiosity	Honesty	Zest	
	Social intelligence		Bravery		Perspective	Modesty		
	Perspective		Love of learning					
	Bravery							
Factor	*Strengths of restraint* (Conscientiousness)	*Strengths of restraint* (Temperance)	*Strengths of restraint* (Cautiousness)	*Strengths of restraint*	*Strengths of restraint/Emotional strengths* (Self-assurance strengths)		*Strengths of restraint*	*Restraint*
Content	Self-regulation	Fairness	Prudence	Self-regulation	Persistence		Persistence	Prudence
	Persistence	Forgiveness	Self-regulation	Persistence	Self-regulation		Self-regulation	Persistence
	Judgment	Modesty	Persistence	Prudence	Hope		Honesty	Self-regulation
	Honesty	Prudence	Spirituality	Perspective	Spirituality		Judgment	Judgment
	Prudence		Honesty	Honesty	Zest		Prudence	Honesty
							Perspective	
							Bravery	
Factor		*Theological strengths* (Transcendence)		*Theological strengths*	*Theological strengths*		*Theological strengths*	*Theological*
Content		Gratitude		Spirituality	Gratitude		Spirituality	Zest
		Hope		Gratitude	Love		Gratitude	Hope
		Spirituality		Beauty	Beauty		Beauty	Gratitude
		Zest			Forgiveness		Hope	Spirituality
								Love

*When the names that were originally provided by the researchers differ from those proposed by Peterson and Seligman (2004), they are reported in parentheses*.

### Current study

To better understand the focus of the current study, we must first point out some limitations of studies that have been conducted to date upon which we build our empirical examination of the structure of character strengths. First, all of the studies used a variable-centered approach. This is the dominant approach in the field of personality psychology, which focuses on the associations between the various personality characteristics. It is based on the assumption that the studied group is homogeneous–variables are connected to each other in the same way for all people and no subgroups of different relationship patterns can be distinguished from the studied group (Von Eye and Bogat, 2006). Second, studies on the structure of character strengths are limited to a few countries, and several studies included student samples exclusively. Third, almost all research used the same character strengths measure (i.e., VIA-IS—Values in Action-Inventory of Strengths questionnaire; Peterson and Seligman, 2004) except for Macdonald et al. (2008) who used the IPIP-VIA (International Personality Item Pool-Values in Action). The IPIP-VIA was developed in the International Personality Item Pool (IPIP) project (Goldberg et al., 2006; McCord, 2017). The measurement instruments in the IPIP project are, on the one hand, “proxy” measures (McCord, 2017) because the items were selected from the public domain item pool based on the correlations with the original scales. However, on the other hand, the IPIP-VIA includes both direct and reversed items while the VIA-IS questionnaire contains only direct items. Using different measurement instruments is important in the process of deciding if our current knowledge of character strengths is the knowledge of the strengths themselves or of the tool that measures them. To date, only Macdonald et al. (2008) utilized this questionnaire but in a very small sample (123 psychology freshmen).

The main objective of this study, therefore, is to address the three limitations mentioned above. First, we conducted our analyses using two approaches: the commonly used variable-centered approach and the person-centered approach, which has not been used to analyze character strengths to date. Following the variable-centered approach, we conducted analyses at the scale level (in line with all of the researchers in this field) and the item level (only performed by McGrath, 2014). For the person-centered approach, we were interested in identifying possible types of people similar to each other in terms of configurations of the character strengths that they possess. The person-centered analysis divided the sample into subgroups of individuals who were similar to each other and, at the same time, different from individuals in other subgroups based on their character strengths profiles (Muthén and Muthén, 2000). As such, we were able to describe how character strengths coexist in different types of people. The variable-centered and person-centered approaches complement each other. The variable-centered approach operates on a high level of generality and reveals the connection between character strengths, while the person-centered approach identifies subgroups of people who share the same character strength profile (Laursen and Hoff, 2006). Second, our study was conducted in a large heterogeneous sample of adults rather than in a sample of college students, which is sometimes used to examine the structure of character strengths. The study was conducted in Poland and is the first study on character strengths in Central Europe. Third, we conducted our study using the less applied IPIP-VIA questionnaire that is another measure for assessing character strengths^1^.

We formulated three hypotheses. (1) The structure of four or five factors (presented in Table 2) will be replicated, despite using a different measurement instrument, in a heterogeneous group of adults in a country where similar studies do not exist. We believe that the structure of character strengths is stable regardless of the instrument or population. (2) The item-level analysis may lead to a position that differs from a 24-factor solution. However, analyses that are performed on the extracted factors should lead to a four- or five-factor solution, consistent with McGrath's (2014) findings with a different measure of character strengths. This is expected because some of the 24 scales will be closely related to each other and will form joint factors. (3) By using latent class analysis, types of people who differ in their sets of possessed character strengths will be differentiated. Because there is no data on character strength profiles, we have no expectations for the number and characteristics of these types of people; therefore, we have formulated a research question—what are the characteristics of every revealed character strengths profile?

## Method

### Measurement

We used Najderska and Cieciuch's (2013)^2^ Polish adaptation of the IPIP-VIA questionnaire (Goldberg, 1999; Goldberg et al., 2006)^3^. It was developed as part of the IPIP project. The idea of the project was to create a set of items that measure various personality traits and are freely available according to the principles of open access. From a total of more than 3,200 items, those that fulfilled the criterion of correlation with the original scale were selected. The IPIP-VIA consists of 213 items that comprise 24 scales (there are between 7 and 11 items in each scale) to measure 24 character strengths. Unlike the VIA-IS questionnaire (Peterson and Seligman, 2004), this instrument includes not only direct items (e.g., “Find the world a very interesting place” in the curiosity scale) but also reversed items (e.g., “Hold grudges” in the forgiveness scale). Respondents respond to the statements on a five-point Likert scale ranging from 1 (*very inaccurate*) to 5 (*very accurate*).

All of the IPIP-VIA scales had satisfactory (or boundary, but still acceptable for the research purposes) reliability (Najderska and Cieciuch, 2013). Cronbach's alpha coefficients ranged from 0.66 for the prudence scale to 0.88 for the spirituality scale, with a mean reliability of 0.74.

### Participants and procedure

This study included *N* = 908 respondents (64.1% women and 35.9% men) aged between 18 and 78 years (*M* = 28.58; *SD* = 12.76). The survey was conducted in a paper-and-pencil form by student research assistants who were trained for this task. Each research assistant surveyed between 4 and 10 adults among their family members, acquaintances, or work colleagues. Participation was voluntary, and anonymity was guaranteed. Respondents did not receive compensation for participating in this study.

The IPIP-VIA was one of six research tools used in the larger research project. Conditions of filling in the questionnaires were not standardized. Respondent who gave at least 90% of the same answers or had more than 10% missing data were excluded from further analyses. Regarding the sample size, we asked the research assistants to survey as many people as they possibly could and to include respondents who differed in age and gender.

## Results

### The variable-centered approach: scale level

The first factor analysis included the 24 character strength scales. Parallel analysis (Hayton et al., 2004) was performed using Mplus 7.11 (Muthén and Muthén, 1998–2012), and it suggested a four-factor solution, as did the scree test and eigenvalues. Therefore, we conducted an exploratory factor analysis (with principal axis factoring and oblimin rotation) with four factors.

Table 3 presents the results of the oblimin rotation, as we did not expect that the factors would be orthogonal, and this rotation was used in previous analyses (McGrath, 2014)^4^. Table 3 indicates the factor solutions for the pattern and structure matrices. Only three scales (honesty, kindness, and gratitude) are located on different factors depending on the matrix type. Perspective loadings were only above 0.40 in the structure matrix.

**Table 3:** The four-factor structure of the character strength scale from the IPIP-VIA questionnaire in poland (*N* = 908).

	**Factor**							
	**Emotional/interpersonal strengths**		**Interpersonal/theological strengths**		**Strengths of restraint**		**Intellectual strengths**	
	**Pattern**	**Structure**	**Pattern**	**Structure**	**Pattern**	**Structure**	**Pattern**	**Structure**
Teamwork	**0.76**	**0.67**						
Love	**0.71**	**0.72**						
Leadership	**0.67**	**0.72**						0.42
Humor	**0.66**	**0.68**						0.43
Social intelligence	**0.55**	**0.69**						0.55
Zest	**0.50**	**0.70**				0.43		0.60
Hope	**0.49**	**0.66**						0.53
Bravery	**0.45**	**0.56**	−0.42					0.48
Honesty	**0.45**	0.55		0.52		**0.56**		
Perspective		**0.59**				0.45		0.58
Fairness		0.43	**0.64**	**0.73**		0.44		
Modesty			**0.58**	**0.63**				
Forgiveness			**0.58**	**0.59**				
Kindness	0.48	**0.62**	**0.52**	0.57				0.44
Prudence			**0.51**	**0.63**	0.49	0.59		
Gratitude		**0.58**	**0.48**	0.53				0.51
Spirituality			**0.43**	**0.47**				
Persistence					**0.77**	**0.78**		
Self-regulation					**0.67**	**0.65**		
Judgment					**0.52**	**0.63**		0.42
Love of learning							**0.79**	**0.76**
Curiosity		0.63					**0.64**	**0.80**
Beauty							**0.61**	**0.60**
Creativity		0.55	−0.42				**0.53**	**0.67**

*Loadings below 0.40 are not shown. Bold indicates the highest loadings of each scale*.

The factors that were obtained in this Polish study overlap with the five-factor structure that has been reported in the literature (Peterson and Seligman, 2004; Peterson et al., 2008; Ruch et al., 2010; Singh and Choubisa, 2010; Littman-Ovadia and Lavy, 2012; McGrath, 2014). Two factors are the same: the third factor, which is similar to the category labeled strengths of restraint, and the fourth factor, which is similar to the category labeled intellectual strengths. Although the first factor consisted of character strengths, such as love, humor, zest, and hope (which are connected to the emotional strength factor), it also contains strengths such as teamwork and leadership (which are connected with the interpersonal factor). The second factor is composed of scales that are connected to the interpersonal (including kindness, modesty, and forgiveness) and theological factors (including gratitude and spirituality). Compared to studies from other countries (Macdonald et al., 2008; Peterson et al., 2008; Ruch et al., 2010; Littman-Ovadia and Lavy, 2012; McGrath, 2014), prudence typically loads on the strengths of restraint factor, but the scale description suggests that it is also related to the interpersonal factor, because it results in behaviors such as not saying or doing things that one may later regret, which are also an important aspect of social relationships. In summary, the factors in this study are internally coherent and similar to those obtained in prior research in a way that they can be described using Peterson and Seligman's (2004) logic and terminology. Interpersonal character strengths merge with both emotional and theological strengths.

### The variable-centered approach: item level

The factor analysis at the item level included all 213 items. A parallel analysis that was performed in Mplus 7.11 indicated a 17-factor solution. We conducted exploratory factor analysis with principal axis factoring and oblimin rotation, which was similar to the strategy employed in the above analysis at the scale level. The entire report, with all items loading on factors, can be obtained from the first author upon request. Table 4 presents the number of items from each original scale that loaded onto the factors that were generated by the item-level EFA (with oblimin rotation for the pattern matrix). This analysis provides insight into the meaning of the scales.

**Table 4:** The number of items from the original scales located in the 17-factor solution from an exploratory factor analysis with all IPIP-VIA items.

**Original scales**	**Factors obtained from the item-level EFA**																
	**1**	**2**	**3**	**4**	**5**	**6**	**7**	**8**	**9**	**10**	**11**	**12**	**13**	**14**	**15**	**16**	**17**
Love	6		2									1					
Gratitude	3				1			2					1				1
Zest	2	1	1	1				3				1					
Bravery		5						2							1	2	
Creativity		5				1			2								
Leadership			5									2					
Teamwork			5									4					
Social intelligence			2						2	2			1				
Persistence		1		5												2	
Spirituality	1				8												
Love of learning			1			9											
Curiosity						7		3									
Forgiveness							9										
Kindness	1						4					2	1				2
Hope	1					1		5								1	
Humor								1	8								
Perspective								1		6						2	
Fairness							3				5				1		
Honesty	1		1				1	1			5						
Modesty							1				5		1			2	
Beauty													7				1
Self-regulation				3										5	3		
Judgment										2					6		1
Prudence		1		1			1						1		4		1
Number of items	15	13	17	10	9	18	19	18	12	10	15	10	12	5	15	9	6

The newly obtained scales had satisfactory or boundary (but still acceptable for scientific research purposes) reliability (measured by Cronbach's alpha) and ranged between 0.64 and 0.88 with an average of 0.79. Several newly obtained factors were internally cohesive. One of the best examples is factor 5, which consists of eight items from the original spirituality scale and one item from the original gratitude scale (“Feel a profound sense of appreciation every day”), which, especially in the Polish adaptation, can either be connected to gratefulness, but can also refer to a higher power which one is grateful to. Another example of a cohesive scale is factor 14, which exclusively contains the self-regulation items. However, some scales could not be easily interpreted. For example, factor 17 contains one or two reversed items from the original scales, including gratitude (“Do not see the need to acknowledge others who are good to me”), kindness (e.g., “Get impatient when others talk to me about their problems”), beauty (“Fail to notice beauty until others comment on it”), judgment (“Don't think about different possibilities when making decisions”), and prudence (“Avoid activities that are physically dangerous”). Thus, this scale was inconsistent and could be interpreted as a kind of method effect (because the factors consisted of reversed items) rather than as a specific character strength.

Next, we conducted an EFA (with oblimin rotation) of the 17 factors that were obtained. Again, a parallel analysis suggested a four-factor solution. The results of this analysis are presented in Table 5 (negative loadings are due to reversed items).

**Table 5:** An EFA of the obtained 17 factors.

**Factors**	**Second-order factors**							
	**1**		**2**		**3**		**4**	
	**Pattern**	**Structure**	**Pattern**	**Structure**	**Pattern**	**Structure**	**Pattern**	**Structure**
2	0.82	0.83						
9	0.63	0.71						−0.48
8	−0.51	−0.61		0.48				0.41
6	0.50	0.63						−0.49
10	−0.49	−0.59						0.40
7			0.68	0.71				
13		0.40	0.65	0.68				
5			0.52	0.53				
11			−0.51	−0.62				0.43
1		−0.48	−0.50	−0.65				0.62
12		−0.49	−0.48	−0.60				0.54
15				0.51	0.62	0.71		
4					0.61	0.67		
14					0.41	0.38		
17							0.68	0.71
16		−0.44					0.68	0.67
3		0.47					−0.48	−0.61

*Factor numbers represent the factors from Table 4. Loadings below 0.40 are not shown*.

Analysis of the obtained factors suggests that the first factor represents emotions (e.g., bravery, humor, hope, zest) and intellect (love of learning, curiosity); the second factor represents interpersonal relationships (e.g., love, teamwork, leadership, kindness) and spirituality (spirituality, gratitude, forgiveness); and the third factor represents restraint (self-regulation, persistence, judgment, prudence). The interpretation of the fourth factor is problematic because scales 16 and 17 do not interchangeably indicate the measured strengths. However, scale 3 (which reversely loads onto this factor) is clearly connected to teamwork and leadership and may be a hint for further interpretation of the factor.

### The person-centered approach

A latent class analysis (LCA) was performed on the 24 original centered scales. Table 6 provides details of the two-, three-, and four-class solutions.

**Table 6:** Fit indices of the three LCA solutions.

**Number of classes**	**Degrees of freedom**	**Entropy**	**Class size (in %)**	**Akaike information criterion**	**Vuong-Lo-Mendell- Rubin Likelihood ratio test**
2	73	0.796	37/63	27793.255	0.0008
3	98	0.848	21/15/64	27111.010	0.1145
4	123	0.820	7/20/51/21	26698.029	0.6421

**Figure 1:**
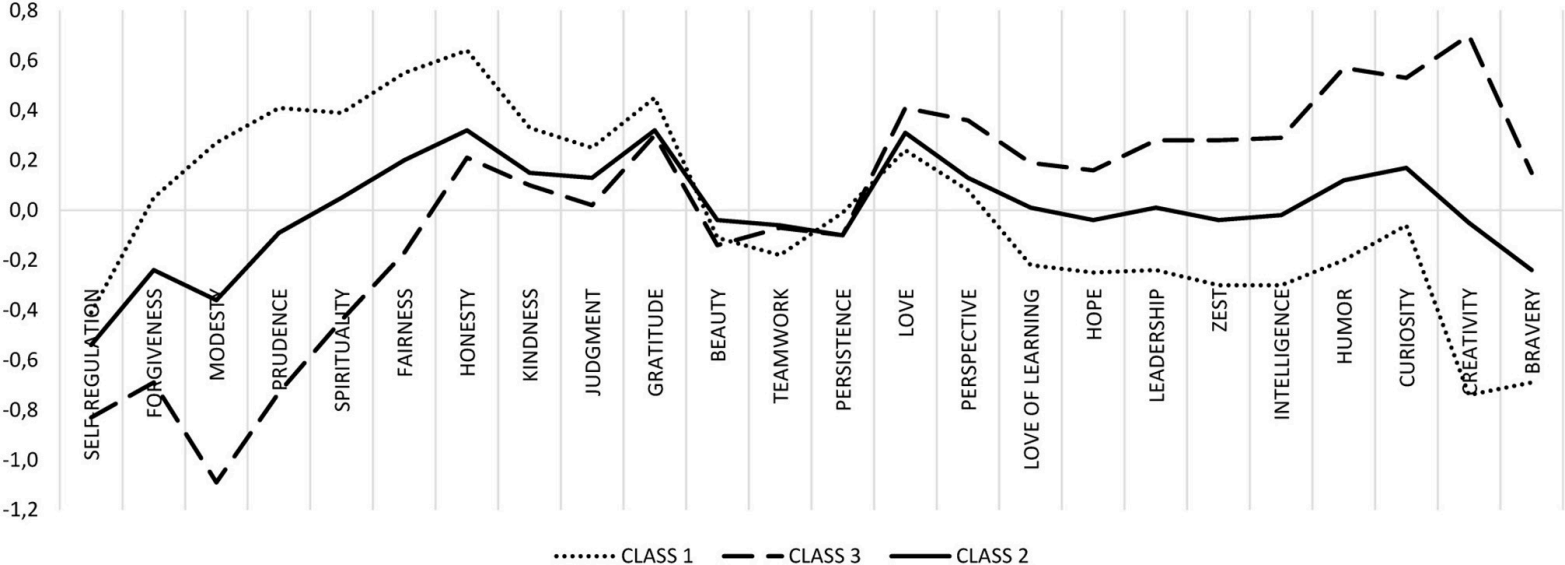
Strengths means for the three-class solution.

To determine the optimal number of LCA classes, we used several fit indices (Merz and Roesch, 2011). The Akaike information criterion and the Vuong-Lo-Mendell-Rubin likelihood ratio values suggest that the four-class solution is the best fit for the data (Merz and Roesch, 2011), while the entropy level suggests that a solution with one class less is optimal. In the four-class solution, one class is very small (7%), so we tested a three-class solution (Merz and Roesch, 2011). The character strength means for each class in this solution are presented in Figure 1, which illustrates that there are three profiles of people for character strengths. Profiles 1 and 3 are symmetrical in comparison to profile 2 (which has average, i.e., between profiles 1 and 3, scores on almost every scale). Profile 1 describes people who score high on honesty, fairness, gratitude, prudence, spirituality, kindness, modesty, and judgment. It was displayed by 21% of the respondents. Profile 3 describes people who score high on creativity, humor, curiosity, love, perspective, social intelligence, leadership, zest, love of learning, hope, and bravery. It was displayed by 64% of the respondents. Profile 2 was displayed by 15% of participants.

## Discussion

The current study extended the knowledge in the extant literature on the structure of character strengths in three ways: (1) by using a different instrument to study character strengths, (2) by conducting studies in a heterogeneous Polish sample, and (3) by using both variable-centered (on scale and item levels) and person-centered approaches.

To test our hypotheses, the first analyses were on the scale level. These analyses led to a four-factor solution in which all factors closely aligned with Peterson and Seligman's (2004) classification system and are also related to the Big Five personality traits. The emotional/interpersonal factor is similar to extraversion, the interpersonal/theological factor is similar to agreeableness, strengths of restraint are similar to conscientiousness, and intellectual strengths are similar to openness. Although similar structures have been obtained in other countries, there are several notable differences. In this Polish study, Factor 1 reflects strengths that usually load onto two factors: emotional and interpersonal (e.g., teamwork, love, leadership, humor, zest, or bravery). Research in other countries (e.g., Peterson et al., 2008; Ruch et al., 2010; McGrath, 2014) connects these strengths only with interpersonal relationships (teamwork, leadership, kindness). In this study, factor 2 merged character strengths from two domains: interpersonal relationships (e.g., forgiveness, kindness, fairness, modesty) and spirituality (spirituality, gratitude), while in most other empirical research these factors are identified as separate. Factors 3 (strengths of restraint) and 4 (intellectual strengths) are similar to those obtained in research across different countries.

In the present study, differences in the factor structure may be due to the specificity of the Polish sample; however, it is also highly possible that they result from using a different measurement instrument, the IPIP-VIA questionnaire (and not the commonly used VIA-IS). This argument is strengthened by comparing our results with those obtained by Macdonald et al. (2008), who also used the IPIP-VIA. In their research, the niceness factor is almost exactly the same as the interpersonal/theological strengths factor in our study (consisting of strengths including modesty, fairness, kindness, forgiveness, spirituality, and gratitude; the Polish sample also includes prudence). Additionally, the positivity factor in their research is very similar to our emotional/interpersonal strengths factor but it contains fewer scales: teamwork, love, hope, humor, zest, and leadership; the Polish sample also included social intelligence, bravery, honesty, and perspective.

To test whether the item-level analysis could lead to a solution other than the 24-factor solution, we performed parallel analysis and EFA with oblimin rotation for all 213 items. The parallel analysis indicated that there are 17 factors (not 24, as suggested by Peterson and Seligman, 2004) that are mostly easy to interpret (except for scales 16 and 17, which only included reversed items that were not connected to a specific strength). It is noteworthy that McGrath (2014), who used the VIA-IS questionnaire (which does not include any reversed items), also had problems identifying the correct number of interpretable factors (in this case, the parallel analysis suggested more factors, but he reduced it to 24 and came up with several scales that could not be named using the terms from the original taxonomy, e.g., receptivity). After performing an EFA on the newly obtained factors, we also found a four-factor structure, which corresponds to the factors that were obtained at the scale level. However, there were also several noticeable differences. The strengths that were related to interpersonal relationships and spirituality were connected with each other in both classifications. In addition, the strengths of the restraint factor were distinguished at both item and scale levels, although emotional and intellectual strengths merged into one factor at the item level. This suggests that emotional strengths, including bravery, humor, hope, or zest, are closely related to intellectual strengths, such as curiosity, love of learning, and perspective, in our study. This was the first and the largest factor in the analysis, thus, social desirability may have affected these results. This interpretation for the first factor obtained in an EFA on personality data was also used by Verkasalo et al. (2009) in their research on values.

The third finding in our study was derived from the use of a person-centered approach. We identified three interpretable profiles of people. Profile 1 depicts people who score high in honesty, fairness, kindness, judgment, spirituality, prudence, and modesty. These are strengths that are connected to interpersonal relationships and to being a part of the society/social group. We propose to call this profile *socialization*. Profile 3 describes people who score high in love, curiosity, perspective, humor, love of learning, leadership, social intelligence, hope, zest, creativity, and bravery. These strengths are connected to growth, personal development, intellect, and the possession of specific social skills. As such, we propose to call this profile *personal growth*. Profile 2 identifies people who do not have a specific set of dominant character strengths because all of their strengths are neither high nor low but average.

Profiles 1 and 3 correspond to personality metatraits (Digman, 1997; DeYoung, 2006, 2010). Metatraits were identified by Digman (1997) but were also later described by DeYoung (2006, 2010). They are higher-order factors built upon the five basic personality traits (neuroticism, extraversion, openness, conscientiousness, and agreeableness) and are responsible for the intercorrelations between these traits (Digman, 1997; Strus et al., 2014; Cieciuch and Strus, 2017; cf. Strus and Cieciuch, 2017). The first metatrait, which is referred to as *alpha* or *socialization* by Digman (1997), is composed of the shared variance of three of the five traits from the Big Five model—agreeableness, conscientiousness, and emotional stability (the opposite of neuroticism). The second metatrait, which is referred to as *beta* or *personal growth* by Digman (1997), reflects the shared variance of extraversion and openness.

On the content level, there is a similarity between the character strengths' profiles and metatraits. Profile 1 corresponds to alpha because it describes people who possess several interpersonally desirable character strengths. They are honest, fair, grateful, prudent, and believe in a higher power. Profile 3 corresponds to the beta dimension and characterizes people who strive for growth. They are creative, curious, and have a good sense of humor but are also loving, intelligent (emotionally, socially, and personally) and have perspective. Our interpretation is in line with the model of metatraits proposed by Strus et al. (2014) who showed that alpha and beta have much broader meaning than those extracted from the Big Five traits and can serve as a frame of reference for interpretation of many psychological constructs.

Peterson and Seligman (2004) described character strengths as “traitlike” constructs, which suggests that they were using a trait paradigm. In our studies, we used both trait (the variable-centered approach) and type (the person-centered approach) paradigms. These two approaches are combined in the field of metatraits, as there is no conflict between them, since both personality types and metatraits are described as a configuration of traits as was shown by Strus et al. (2014).

### Limitations

The results presented in this article are the first obtained with the Polish version of the IPIP-VIA. Replication of the analysis is definitely the next step in verifying the structure of both factors (on scale and item levels) and profiles. A comparison with the results of the Polish version of VIA-IS would also be very helpful; however, the results of this adaptation are not available yet. In addition, it would be worthwhile to control the social desirability in future research, since it can certainly play a role in the results due to the procedure we used in our study and to the positive character of the measured variables (respondents usually tend to describe themselves as above average when it comes to socially valued traits).

The relationship between character strengths and personality are not yet clear. Peterson and Seligman (2004) proposed several theoretical connections between strengths and factors from the Big Five theory. Nevertheless, this is a topic that requires further research. A more in-depth examination of character strengths, including the factors they group onto and the profiles they create, may provide a starting point for locating them in the structure of personality.

Despite the similarities between strengths and personality metatraits, this relationship is purely theoretical and is solely based on the correspondence of these constructs. Further research should measure both constructs (i.e., character strengths and personality metatraits) to determine whether this relationship has empirical support.

## Ethics statement

This study was carried out in accordance with the recommendations of ethic board of Cardinal Stefan Wyszyński University in Warsaw with informed consent from all subjects. The protocol was approved by the ethic board of Cardinal Stefan Wyszyński University in Warsaw. Participants were adult and participation in the study was voluntary. Only oral consents were required in this study, which is an approved procedure in our university in questionnaire research on adults.

## Author contributions

MN: Data collection, data analysis and interpretation, drafting the article, and final approval of the version to be published. JC: Conception or design of the work, data analysis and interpretation, critical revision of the article, and final approval of the version to be published.

### Conflict of interest statement

The authors declare that the research was conducted in the absence of any commercial or financial relationships that could be construed as a potential conflict of interest.

## Footnotes

^1^The data, syntaxes, and any other documents available upon request from the first author.

^2^The analyses presented in this paper were performed on the data collected for the purpose of validating the IPIP-VIA questionnaire.

^3^Original IPIP-VIA items are available on the www.ipip.ori.org website.

^4^We used both oblimin and varimax rotations, and the results were quite similar. Very few scales were placed in different factors for the two types of rotation, e.g., creativity in oblimin rotation is located on the intellectual strengths factor, while it is located on the emotional/interpersonal strengths factor in varimax rotation.
